# Diversity, Equity, and Inclusion of Dermatology Journals and Their Editorial Board Members

**DOI:** 10.2196/44217

**Published:** 2023-03-10

**Authors:** Julianne Kiene, Sarah Minion, Ramiro Rodriguez, Robert Dellavalle

**Affiliations:** 1 School of Medicine Georgetown University Washington, DC United States; 2 College of Medicine The University of Iowa Carver Iowa City, IA United States; 3 Department of Dermatology School of Medicine University of Colorado Denver, CO United States

**Keywords:** diversity, equity, inclusion, dermatology

## Abstract

Dermatology as a whole suffers from minority underrepresentation. We conducted a search of the top 60 dermatology journals for mention of their approach to increasing diversity, equity, and inclusion (DEI) within their publication through editorial board members or peer-review processes. Of those 60, only 5 had DEI statements or editorial board members dedicated to increasing DEI. There are publications with checklists and frameworks for increasing DEI within the literature. We propose that more journals implement these resources within their peer-review process to increase diversity within their publication.

Research journals with diverse editorial board members are more likely to publish research from diverse perspectives [1]. As the dermatologic specialty suffers from minority underrepresentation [2], it is pivotal that dermatology journals strive to achieve diverse editorial boards and peer reviewers. Only one study has explored the racial and ethnic demographics of two high-impact dermatology journals, *JAMA Dermatology* and the *Journal of the American Academy of Dermatology* (*JAAD*), finding that their racial demographics mirror that of dermatology, although unrepresentative of the United States population [3]. To the authors’ knowledge, no other studies have explored the diversity of other dermatology journal editorial board members. Improving the diversity, equity, and inclusion (DEI) of academic dermatology requires acknowledgment, leadership roles, and future directions to broaden representation, reduce bias, and improve health disparities. Thus, our goals were to identify DEI efforts of top dermatology journals; to recommend resources for dermatology journal DEI improvement; and to propose *JMIR Dermatology*’s efforts to improve DEI in our peer-review process.

We identified the 60 highest-impact dermatology journals on Scimago by their h-index score. Each journal’s website was examined for statements on DEI, editorial board members dedicated to DEI, or other information regarding DEI in their peer-review process. Two independent reviewers performed website searches and documented the results separately to improve accuracy. If the journal was a subsidiary of an academic society, we did not include diversity statements made by the society unless it was explicitly stated on the journal’s website.

Of the 60 dermatology journals reviewed, only 5 (8%) referenced DEI either as a policy on its impact on publication processes or identified individuals or groups dedicated to increasing the diversity of published research. For example, *JAMA Dermatology* has an associate editor for diversity, a mission statement, and mentorship initiatives aimed at advancing DEI within publications. Further, *JAAD* and the *British Journal of Dermatology* have editorial groups dedicated to DEI. *Pediatric Dermatology* has a DEI statement. Finally, *Actas Dermo-Sifiliograficas* has listed the gender diversity of editors. No other journals met our inclusion criteria. The JAMA Network and American Society of Nephrology created editorial policies and a DEI checklist, respectively, to provide a framework and criteria to ensure DEI is an integral part of the editorial and peer-review processes [4-6]. The recommendations by these journals can be used as a resource for dermatology journals, editors, and peer reviewers desiring to increase DEI in their publications.

*JMIR Dermatology* is dedicated to improving the DEI within our publications and our peer-review process. We plan to appoint a DEI editor to our editorial board who will lead a diverse editorial review committee. The DEI committee will provide manuscript feedback, recommendations, and guidance to encourage diverse author representation and ensure the use of inclusive language. We urge dermatology journals to join in this step toward improving racial and ethnic DEI in journal review boards.

## Abbreviations

DEI: diversity, equity, and inclusion
JAAD: Journal of the American Academy of Dermatology
